# Effectiveness and safety of Daixie Decoction granules combined with metformin for the treatment of T2DM patients with obesity: study protocol for a randomized, double-blinded, placebo-controlled, multicentre clinical trial

**DOI:** 10.1186/s13063-023-07301-4

**Published:** 2023-04-19

**Authors:** Zhidong Liu, Kepei Zhang, Jianpin Zhang, Fei Wang, Yang Zhou, Lin Wang, Miao Wang, Yi Zhang, Shigao Zhou

**Affiliations:** 1 Department of Internal Medicine of Traditional Chinese Medicine, Longhua Hospital, Shanghai University of Traditional Chinese Medicine, 725 South Wanping Road, Shanghai, 200032 China; 2 Department of Traditional Chinese Medicine, Shanghai Jinshan Hospital of Integrated Chinese and Western Medicine, Shanghai, 201501 China; 3 Department of Traditional Chinese Medicine, Yueyang Hospital of Integrated Chinese and Western Medicine, Shanghai University of Traditional Chinese Medicine, Shanghai, 200437 China; 4 Department of Traditional Chinese Medicine, Shanghai Pudong New District People’s Hospital, Shanghai, 201299 China

**Keywords:** Daixie Decoction granules, T2DM with obesity, Evidence-based clinical trial, Traditional Chinese medicine, Protocol

## Abstract

**Background:**

Type 2 diabetes mellitus (T2DM) with obesity is a glycolipid metabolism disorder, which makes hypoglycaemic treatment more complex and increases the proportion of multidrug combinations. In addition, patients are more prone to adverse reactions and gradually lose compliance with treatment. Previous clinical trials have demonstrated that Daixie Decoction granules (DDG) can reduce body weight and blood lipids and improve the quality of life of T2DM with obesity. But there are a lack of further evaluations for the efficacy and safety of DDG combined with metformin.

**Methods/design:**

The study is designed as a multicentre, randomized, double-blind, placebo-controlled clinical trial. Participants who meet the Nathrow criteria will be randomly assigned to the intervention group and control group (*n*_1_ = *n*_2_ = 133). Based on a unified diet control and exercise therapy, the intervention group will be treated with DDG and metformin, and the control group will be treated with DDG placebo and metformin. All subjects will receive a 6-month treatment followed by a 6-month follow-up. Effective rate of a 1% decrease in HbA1c and 3% decrease in body weight will serve as the primary outcome. The secondary outcome include fasting plasma glucose, blood lipids, C-peptides, insulin, inflammatory factors, insulin resistance index (HOMA-IR) and the subcutaneous and visceral fat content in the upper abdomen measured by MRI. Blood routine, urine routine, stool routine, liver and kidney function, EKG and other safety indicators and major adverse reactions were monitored during total treatment and follow-up time.

**Discussion:**

We aimed to determine the efficacy and safety of DDG combined with metformin for the treatment of T2DM patients with obesity.

**Trial registration:**

Trial registration: ChiCTR, ChiCTR2000036290. Registered 22 August 2014,  http://www.chictr.org.cn/showprojen.aspx? proj=59001

## Strengths and limitations of this study

- Previous exploratory studies have confirmed that Daixie Decoction granules can reduce plasma glucose, body weight and blood lipids.
- Based on a previous clinical study, the clinical effect of Daixie Decoction granules combined with metformin in the treatment of T2DM with obesity will be observed using a multicentre randomized controlled double-blind method.
- Randomization and arrangement to distribute drugs will be applied by the centre, and the placebo will be prepared in uniform by manufacturer of Daixie Decoction granules to ensure that researchers and patients do not have direct contact with the drug preparation and delivery process.
- We will objectively evaluate the efficacy of the intervention based on the aspects of blood lipids, blood sugar, insulin resistance and chronic inflammatory state and comprehensively analyse the benefits of intervention for type 2 diabetes patients with obesity.
- Our experiment will be conducted in four centres in the same region of China, and it is uncertain whether similar effects will occur in other regions and races.

## Introduction

In the past 30 years, China’s economy has developed rapidly. The change in people’s lifestyle to a sedentary lifestyle and high-energy/high-fat diet has directly led to an increase in the number of people with type 2 diabetes and obesity [1]. The prevalence of type 2 diabetes among adults in China has increased rapidly from 0.67% in 1980 to 10.9% in 2013, making it the country with the largest number of diabetic patients. It is estimated that diabetes-related medical costs in China reached $110 billion in 2017, representing a huge economic burden [2].

The prevalence of obesity in China increased from 5.7% in 2010 to 6.3% in 2017, and waist circumference also increased significantly from 80.2 cm in 2007 to 83.2 cm in 2017 [3]. Weight gain is an independent risk factor for T2DM [4]. Studies have suggested that increased body weight or waist circumference can aggravate insulin resistance and increase the risk of T2DM and the difficulty of plasma glucose control [5-7]. Compared with those with simple obesity, people with T2DM and obesity have a more difficult time losing and maintaining weight. First, insulin inhibits fat decomposition and promotes fat synthesis, and insulin levels in obese patients increase significantly [8]. Second, the synergistic effect of obesity and T2DM in other metabolic abnormalities can aggravate insulin resistance in T2DM, and the increase in visceral fat may represent the main cause of insulin resistance in obese patients [9]. Weight loss can improve insulin resistance and reduce plasma glucose and risk factors for cardiovascular disease. Controlling weight is an important intervention strategy for T2DM complicated with obesity [10].

Hypoglycaemic therapy for T2DM with obesity is also more complex [11]. Obesity-induced insulin resistance makes it difficult to reach the plasma glucose standard. If improper treatment leads to continuous weight gain, insulin resistance will be further aggravated, so it is necessary to increase the dosage or other types of drugs accordingly to maintain the stability of plasma glucose [12]. When a greater proportion of multidrug combinations is used in treatment, adverse reactions are more likely, and patients exhibit reduced compliance with treatment [13]. The purpose of treatment is not only to control plasma glucose but also to prevent complications, improve the quality of life and save lives [14]. The greatest threat to T2DM patients is complications associated with large vessels, namely, coronary atherosclerosis and stroke [15]. Therefore, for T2DM patients with obesity, how to achieve standard plasma glucose levels to avoid macrovascular and microvascular complications, effectively reduce body weight and reduce the occurrence of obesity-related hypertension, hyperlipidaemia and stroke are clinical problems that must be solved.

Traditional Chinese medicine compounds have the characteristics of multicomponent, multilink and multichannel action, have a variety of pharmacological effects and play a therapeutic role in many key links of diseases [16]. DDG has become department cipher prescription based on long time clinical verification (specific prescription: *Atractylodes lancea* 18 g, *Atractylodes macrocephala* 18 g, *Salvia miltiorrhiza* 15 g, *Polygonum cuspidatum* 12 g, Folium ilicis cornutae 15 g, *Scutellaria* 12 g, *Linderae radix* 9 g). In our previous clinical studies, 80 subjects of T2DM with obesity were recruited, and the intervention treatment of traditional Chinese medicine DDG combined with metformin was conducted for 13 weeks using self-controlled experimental study methods before and after. The results showed that after 13 weeks of intervention, patients exhibited increased weight loss; glycosylated haemoglobin, fasting plasma glucose (FPG) and postprandial plasma glucose levels were decreased; triglycerides, low-density lipoprotein and free fatty acid levels were improved; and high-density lipoprotein levels were increased. DDG also improves insulin resistance in patients, reduces body weight and blood lipids while lowering glucose levels, improves the quality of life of patients and plays roles in comprehensive prevention and treatment. More importantly, during the 13-week intervention, no serious adverse effects were detected. And only 2 cases had adverse effects of diarrhea, and all completed the trial. The relevant achievements have been patented (Application No. 201710563974.4) and are now in the stage of substantive examination.

Therefore, based on a multicentre randomized, double-blind clinical study design, this study aims to sufficiently verify that DDG combined with metformin is effective and safe as an intervention in patients who have T2DM with obesity, laying a foundation for providing valuable new drugs for clinical use.

### Objectives

A preliminary clinical study found that DDG reduces body weight and blood lipids while lowering glucose, improves the quality of life of patients and plays roles in comprehensive prevention and treatment. We attempt to further understand and verify the efficacy and safety of DDG in T2DM with obesity through clinical studies and comprehensively evaluate the benefits of this intervention in patients.

## Methods and analysis

### Study design

**Fig. 1:**
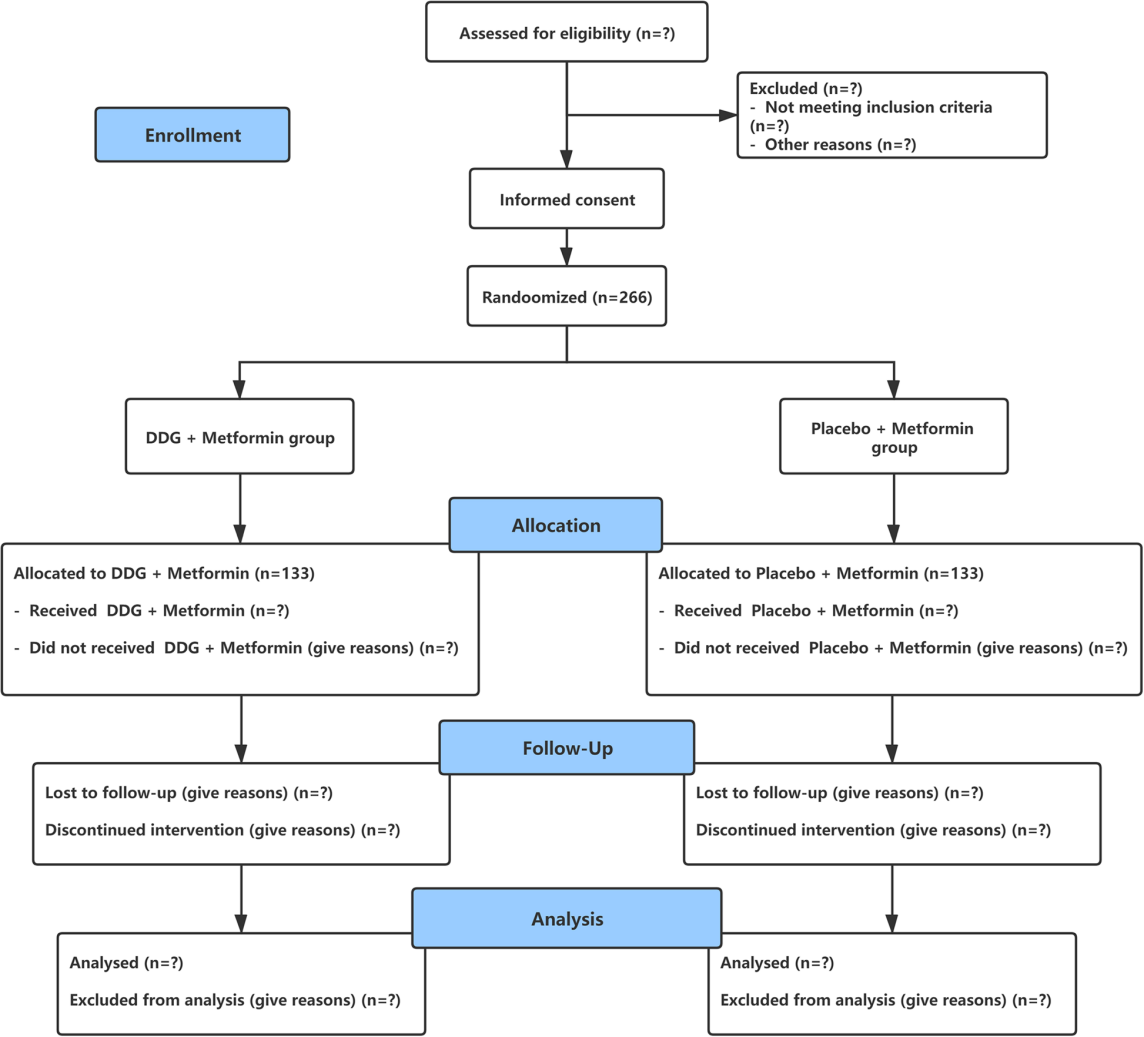
The study flow chart of the whole multicentre RCT

The study is designed as a multicentre, randomized, double-blind, placebo-controlled, parallel design clinical trial in which subjects were randomly assigned in a 1:1 ratio to either an intervention (DDG combined with metformin) or a control group (placebo combined with metformin). The random number assignment will be provided by the Clinical Research Center of Longhua Hospital Affiliated to Shanghai University of Chinese Medicine. Participants in each group will receive 24 weeks of treatment and follow-up for 24 weeks after treatment. The study flow chart is shown in Fig. 1.

**Fig. 2:**
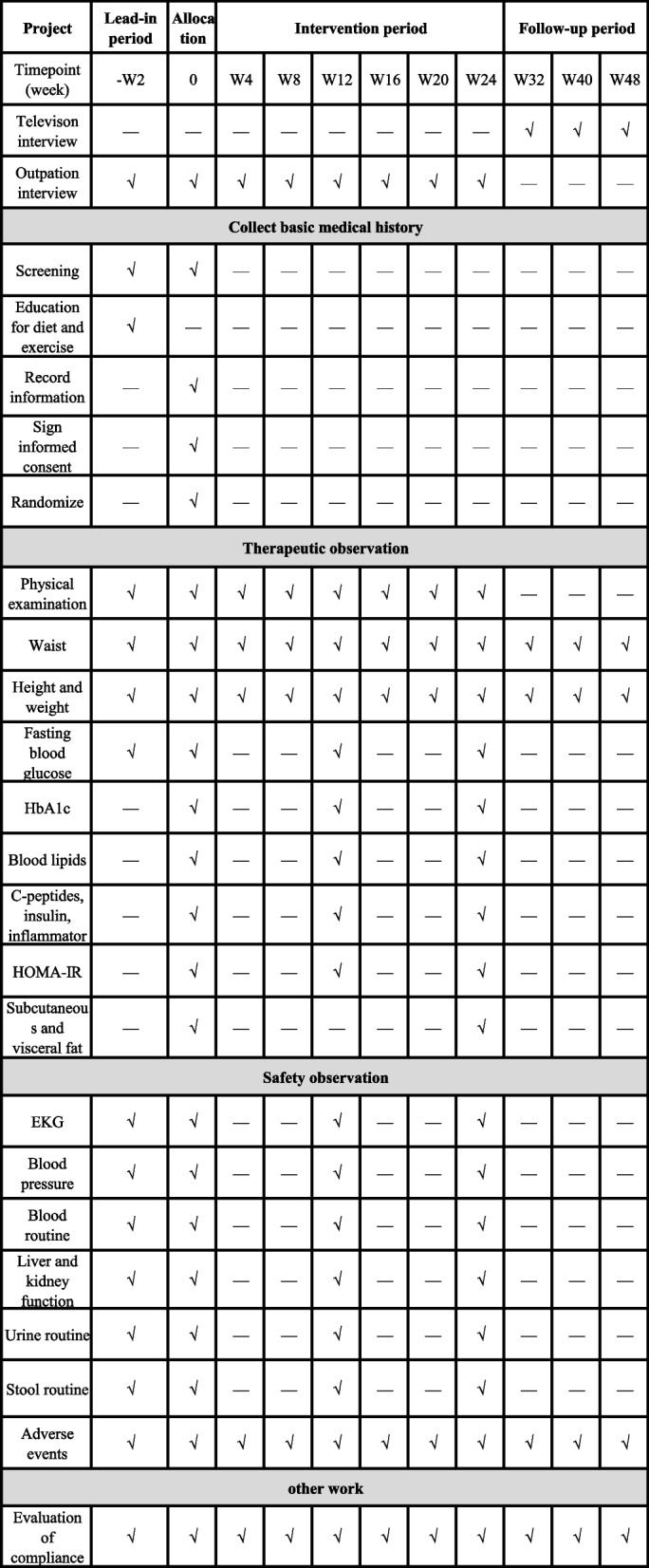
The schedule of participants

We will record all baseline observations involved during the screening period. During the period of treatment, all participants will be required to record BMI and waist values daily. Routine blood tests, routine urine tests, routine stool tests, and blood biochemistry will be performed and recorded every 3 months. Intraperitoneal fat thickness (imaging MRI measurement of fat layer thickness) will be measured and recorded once each before and after treatment. After the end of the last treatment, all patients will enter the posttreatment follow-up period. All subjects will be followed up once a month. The schedule of participants is shown in Fig. 2.

### Subject screening and selection

For outpatients or inpatients who come to the hospital, if the diagnostic criteria for type 2 diabetes with obesity are met (as shown in Tables 1 and 2), the inclusion and exclusion criteria will be assessed (as shown below). Patients will not be recruited if any of the inclusion criteria are not met or if any of the exclusion criteria are met. If the patient meets one of the rejection criteria or termination criteria, they will be removed from the trial. Participants will be required to provide written informed consent to cooperate with the doctor’s treatment and follow-up and to provide information that does not exceed the scope of ethical approval. Participants who did not meet the requirements will be removed from the study. During this period, medical history, physical examinations, blood samples and medical records of participants will be collected and kept.

**Table 1:** Diagnostic criteria for type 2 diabetes [13]

Standard of diagnosis	Glucose levels in intravenous plasma mmol/L
Typical diabetes symptoms (polydipsia, polyuria, polydipsia, weight loss) plus random plasma glucose testing	≥ 11.1
Fasting plasma glucose test	≥ 7.0
Plasma glucose measured 2 h after glucose load Repeat the test another day for no symptoms of diabetes	≥ 11.1

**Table 2:** Diagnostic criteria for obesity [13]

Classification	BMI (kg/m^2^)
- Underweight	< 18.5
- Normal weight	18.5–23.9
- Overweight	24.0–27.9
- Obesity	≥ 28.0
For waist	(cm)
- Male	≥ 90.0
- Female	≥ 85.0

The study aims to recruit 266 participants (4 units in total). The four units include the following major leading and undertaking units. The Longhua Hospital affiliated with Shanghai University of traditional Chinese Medicine, which will recruit 86 participants. The remaining participating units will recruit 60 participants each: Yueyang Hospital of Integrated Chinese and Western Medicine affiliated with Shanghai University of traditional Chinese Medicine; Shanghai Jinshan Hospital of Integrated Chinese and Western Medicine; Shanghai Pudong New District People’s Hospital. Each unit is responsible for the specific responsibilities of the study, including recruiting patients and collecting specimens and data. Laboratory testing and imaging examination will be performed in the corresponding departments of each unit. Data analysis will be performed at the Clinical Research Center of Longhua Hospital affiliated with Shanghai University of Traditional Chinese Medicine.

To ensure the safety of the participants, blinding, data quality, and adherence to the study protocol, personnel directly participating in this study will be completely trained and will follow Good Clinical Practice (GCP) guidelines.

### Inclusion, exclusion and rejection criteria and termination criteria

#### Inclusion criteria

1. Patients 18–65 years old of any sex;
2. Patients who meet the diagnostic criteria of type 2 diabetes and obesity;
3. Patients who had been diagnosed with type 2 diabetes in the past 12 months, and had not taken any anti-diabetic medication before;
4. After 2 weeks of diet control and exercise therapy, patients whose FPG is greater than 7.0 mmol/L but less than 13.9 mmol/L, and the FPG level does not change more than 2.5 mmol/L before and after the treatment;
5. Patients who agreed to participate in this clinical study voluntarily signed informed consent forms and agreed to participate in all visits and examinations as required by the study protocol.

#### Exclusion criteria [11]

1. Patients with nephrotic syndrome (urine protein exceeding 3.5 g/L per day), oedema or renal failure (serum creatinine over 115 μmol/L);
2. Patients who have been diagnosed with heart failure (NYHA III-IV) or who have had a pacemaker implanted;
3. Patients with liver dysfunction (aspartate aminotransferase and glutamate pyruvate transaminase levels twofold above the normal range) or a diagnosis of cirrhosis;
4. Patients with high levels of HbA1C (greater than 9%);
5. Pregnant or breastfeeding mothers, including mothers who have given birth within the last 24 weeks;
6. Patients who require insulin therapy;
7. Patients who receive other therapies or whose doses have changed during treatment;
8. Patients with secondary obesity caused by other diseases, such as Cushing’s syndrome, primary hypothyroidism, hypothalamic obesity, polycystic ovary syndrome;
9. Patients who received weight-loss supplements, antidepressants or hormonal medications during the past 3 months and during the study period;
10. Patients with diabetic ketoacidosis or hyperosmolar non-ketoacidosis coma;
11. Patients with an allergy to intervention drugs;
12. Patients with severe hypertension (SBP>160 mmHg, DBP > mmHg);
13. Patients with acute or chronic pancreatitis or pancreatic insufficiency.

#### Rejection criteria

Those found in the middle of the trial who failed to meet the case selection criteria; failure to complete the diet exercise in accordance with the regulations; failure to use drugs as prescribed, unable to judge efficacy or providing incomplete information; taking drugs that were prohibited and may affect the judgement of the effect.

#### Drop out criteria

Patients who must stop medication because of serious adverse drug reactions; patients who quit the trial due to ineffective treatment; patients who are unwilling to accept treatment or unable to complete follow-up due to poor compliance; patients who have no adverse reactions during treatment, but the treatment is interrupted due to other reasons, such as emigration and missing visits.

#### Termination criteria

Subjects who withdraw informed consent and request withdrawal from the study; pregnancy events occur during the study; side effects of drug or treatment operations are still intolerable; a situation in which the researcher deems it necessary for study withdraw.

If the participant meets the rejection criteria, they are removed, and the data will not be included in the final statistical analysis. However, the termination criteria are related to patients who may terminate during treatment, so the data from these patients will be included in the final statistical analysis.

### Informed consent

When selecting qualified participants, the researcher must describe the details of the clinical trial, including the purpose of the trial, the trial procedure, possible benefits and risks, and the rights and obligations of the subjects, so that the participants can completely understand and have sufficient time to consider. After their questions are answered satisfactorily, participants will sign the informed consent. When each participant signs an informed consent form, the doctor leaves his or her contact number with the participant, so he or she can contact the doctor at any time if conditions change.

### Randomization

After informed consent is obtained, participants will be randomly assigned in a 1:1 ratio to either an intervention (DDG combined with metformin) or a control group (placebo combined with metformin). Randomization will be performed by the Clinical Research Center of Longhua Hospital Affiliated to Shanghai University of Traditional Chinese Medicine.

#### Sequence generation

The method of central stratification and block randomization will be adopted. SAS software will be used to make a random sequence table to generate random numbers as drug numbers.

#### Allocation concealment mechanism

A specific statistical researcher who does not participate in the clinical trial will be responsible for generating the randomization sequence and distribute the number to the experimental products. The observer will remove the envelopes with corresponding serial numbers according to the order of each subject’s visit and distribute the drug according to the drug number, which will remain unchanged throughout the study.

### Blinding

The random distribution system will be implemented and managed by the Clinical Research Center of Longhua Hospital affiliated with Shanghai University of Traditional Chinese Medicine. The randomization code will be maintained by a specially appointed independent custodian. Researchers, participants, inspectors and data inputters will all be blind. The drug will be randomly coded according to the random scheme, and the coding number will become the unique identification code of the participants. According to the order of visits, subjects will be randomly divided into different groups. The dosage form and dose of the tested drug and the placebo are the same, and the drug will be uniformly packaged in the same drug label and distributed by the centre. The DDG placebo is prepared by the same manufacturer to ensure that the researchers and the patients do not have direct contact with the drug preparation and delivery process, so the double-blind method can be guaranteed. All participants and researchers in the trial will be asked to complete a questionnaire to evaluate the treatment at the last visit to assess the success rate of blinding [17].

### Sample size calculation

Our aim is to select an estimation method for sample size when comparing two sample means. When the number of samples is expected to be equal between the test group and the control group, the following formula can be used [18]:

$$n_C=\frac{\left(Z_{1-\alpha}+Z_{1-\beta}\right)^2\sigma^2\left(1+{\displaystyle\frac1K}\right)}{\left(\mu_T-\mu_C-\triangle\right)^2}$$

Here, *n*_*c*_ represents the estimated sample size of each test group, where *μ*_T_ and *μ*_C_ are the mean of the test group and the control group, respectively, and *σ* is the standard deviation (assuming the standard deviation of the two groups is the same). In addition, *α* and *β* are type I errors and type I errors, respectively. *K* is the distribution ratio of the number of cases between the experimental group and the control group. The study is required to have a probability of > 5% I errors and 20% II errors, so *α* = 0.05 and *β* = 0.2 [19]. According to previous studies, *μ*_T_ = 0.85, *μ*_C_ = 0.5, and *σ* = 1.4. Based on the pre-experimental results and an estimated missing follow-up rate of 10%, the final number of cases included is 266.

## Interventions

### Dietary and exercise therapy

After entering the group, all subjects will adopt a unified control diet and exercise regimen that will remain unchanged in subsequent studies. In this study, to control total energy, for T2DM patients with greater than normal body weight, it is recommended to calculate the total energy according to 25 ~ 30 kcal/(kg · day) and then adjust to individual energy standard according to the patient’s height, weight, sex, age, activity, stress status, etc. A long-term very low-calorie diet (less than 800 kcal/day) is not recommended.

To cultivate a nutritionally balanced diet, protein intake should be 15 ~ 20% of total energy, fat intake should be less than 30%, and carbohydrate intake should be 45 ~ 60% of total energy.

Regarding the exercise regimen, at least 150 min of moderate-intensity aerobic exercise per week (50 ~ 70% of the maximum heart rate; maximum heart rate = 220 − age) should be performed for at least 3 days a week.

Please refer to “Expert consensus on comprehensive management of type 2 diabetes with obesity in China” for more information [13].

### Drugs and usage

#### The DDG combined with metformin group

One pack of DDG (*Atractylodes lancea* 18 g, *Atractylodes macrocephala* 18 g, *Salvia miltiorrhiza* 15 g, *Polygonum cuspidatum* 12 g, Folium ilicis cornutae 15 g, *Scutellaria* 12 g, *Linderae radix* 9 g) will be administered twice a day by bringing water to a boil, stirring, covering and sealing for 3 to 5 min before serving.

Metformin will be started at one tablet (0.5 g) once daily after dinner during the first week of treatment, titrated up to a maximum of two tablets twice daily (maximum dose metformin 2000 mg daily) over 4 weeks as tolerated with continuation until the end of treatment. The treatment will be conducted for 24 weeks, and patients will be followed up for 24 weeks.

#### The placebo combined with metformin group

One pack of placebo granules will be administered twice a day by bringing water to a boil, stirring, covering and sealing for 3 to 5 min before serving.

The metformin dose was the same as that in the DDG combined with metformin group.

The treatment will be conducted for 24 weeks and followed up for 24 weeks. Participants will return the unused tablets and bottle at each follow-up visit. Unused tablets will be counted and recorded on the appropriate CRF (case report form). Other hypoglycemic, lipid-lowering or weight-loss drugs were not allowed to be used during the trial.

DDG and placebo granules will be provided by Sichuan New Green Pharmaceutical Technology Development Co., Ltd. Metformin will be manufactured by Sino-American Shanghai Squibb Pharmaceutical Co., Ltd.

## Data collection and management

### Data collection

Data will be collected from the daily symptom records recorded by the participants, the CRF recorded by the medical staff of the trial group and the medical records of the patients. Study data will be collected and managed using REDCap electronic data capture tools hosted at LongHua Hospital Shanghai University of Traditional Chinese Medicine. REDCap (Research Electronic Data Capture) is a secure, web-based software platform designed to support data capture for research studies that provides (1) an intuitive interface for validated data capture, (2) audit trials for tracking data manipulation and export procedures, (3) automated export procedures for seamless data downloads to common statistical packages, and (4) procedures for data integration and interoperability with external sources [20, 21].

### Data entry and data query forms

Data entry and administration are the responsibility of the assigned data administrator, who will establish the database and the validation programme. The data are then entered and confirmed by two professionally trained keyboard operators. After checking the CRF, the identified input errors will be corrected until the input data are identical to the CRF. Any queries on the CRF will be communicated to the investigator via a data query table. These data query tables must be processed, signed and dated by the researcher in a timely manner. The resolved data problem must be returned to the data manager, which will modify and validate the data based on the feedback, update the database accordingly, and then query the necessary data problem table again.

### Database locking

After confirming the accuracy of the established database by blind checking, the database will be locked. The locked data cannot be changed. If any data revisions are required after locking, an official statement signed jointly by the applicant, principal investigator, experimental project manager, statistician and data manager must be provided. In addition, revisions will be made in the statistical analysis [17].

## Evaluation

### Primary outcome

Effective rate of a 1% decrease in HbA1c and 3% decrease in body weight.

### Secondary outcome

Waist circumference, BMI, FPG, blood lipids, C-peptides, insulin, inflammatory factors, insulin resistance index (HOMA-IR)[HOMA-IR = FPG (mmol/L) × FINS (mIU/L)/22.5], and the subcutaneous and visceral fat contents of the upper abdomen will be measured by MRI.

### Security outcome

Routine blood tests, routine urine tests, routine stool tests, liver and kidney function tests, and EKG.

### Adverse events observation and recording

Upon entering the clinical trial, the patient’s vital signs, symptoms and any adverse events of disease or laboratory parameters will be recorded. The occurrence of adverse events is not necessarily related to the study drug. Adverse events were classified into three grades: mild, moderate and severe. Serious adverse events occur when the agent (1) results in death; (2) endangers life; (3) results in hospitalization or prolonged hospitalization; (4) causes deformities or birth defects; (5) causes permanent disability or (6) causes cancer. All serious adverse events were reported to the State Food and Drug Administration, Sponsor and Ethics Committee within 24 h. Any causal relationship between adverse events and the drug under this study will be determined by classification.

If a participant has a serious adverse event, the specific condition of the drug use must be known, and unblinding should only be performed with the consent of the principal investigator. Once reading the emergency letter, the case will be considered shedding.

### Statistical analysis

#### Population of statistical analysis

##### Full analytical set (FAS)

This term refers to the set including finished and shedding cases but excluding rejection cases. This group includes those who has taken at least one medication and were recorded in at least one follow-up.

##### Per-protocol set (PPS)

This term refers to cases that meet the inclusion criteria, did not meet the exclusion criteria and completed the treatment plan. In other words, the cases that meet the trial plan, have good compliance and complete the contents prescribed by the CRF will be analysed. PPS will be used to analyse the main indicators for evaluating efficacy and to assess for conformance with FAS results.

##### Safety analysis set (SAS)

This term refers to actual patients who have received at least one treatment and have safety indicators documented. The missing values of SAS shall not be carried forward. Some cases that could be evaluated were excluded, such as those whose age exceeded the inclusion criteria but not those whose safety could not be determined due to the use of banned drugs. For patients with severe adverse reactions or even death during the trial, the data before the change of treatment or before death will be retained for analysis.

In this trial, FAS will be used to analyse baseline data and efficacy. In addition, the primary efficacy indicators will be analysed using PPS, but the conclusions will mainly be based on FAS analysis. If the results of FAS analysis and PPS analysis are consistent, the credibility of the conclusion will be increased. If data are missing, the data will be extrapolated using the last observation carry-forward (LOCF) method.

### Statistical analysis methods

The data of this clinical trial will be analysed using SPSS Statistics 26.0 statistical software. First, the baseline situation of the two groups of patients will be analysed to ensure that the baseline of the treatment group and the control group are balanced. Normal tests will be performed for measurement data. Continuous variables conforming to a normal distribution will be represented by the mean and standard deviation. Two-independent sample *t*-tests will be used for two groups that conform to a normal distribution, and paired sample *t*-tests will be used for comparison of the same sample before and after comparison. Counting data will be represented by percentages, and the chi-square test will be adopted. For continuous variables that do not conform to the normal distribution, the median will be used, and the independent sample Wilcoxon rank sum test will be used for the two groups. The paired sample Wilcoxon rank sum test will be used for the comparison of the same sample before and after. The Wilcoxon rank sum test will also be used for ranked data. *P* ≤ 0.05 will be considered statistically significant.

## End of trial

The trial will end once 266 participants have been recruited and all have completed 24 weeks of treatment and 24 weeks of follow-up.

## Ethics and dissemination

### Ethical approval and supervision

Prior to entering the study, written informed consent will be obtained from all participants. Voluntary participation will be ensured, and maximum confidentiality of information provided during the interview will be required. The trial will be supervised by the Ethics Committee. Any moderate or serious adverse events will be reported to the Ethics Committee. Participants with adverse reactions during the trial will receive free medical care.

### Confidentiality

All research-related information will be securely stored at the research site. All participant information will be stored in a locked file cabinet in a restricted access area. All laboratory samples, reports, data collection, processes and management forms will be identified by coded ID (identification) numbers only to maintain the confidentiality of participants. All records containing names or other personal identifiers (such as informed consent) will be stored separately from research records identified by code numbers. All local databases will be protected using password-protected access systems. Tables, lists, logs, dating books and any other list linking participant ID numbers to other identification information will be stored in separate locked files in the access-restricted area.

### Patience and public participation

The relevance and necessity of research questions, research design and patient-oriented documents (including informed consent, participant information tables, symptom diaries, all follow-up forms and promotional materials) have been reviewed by the public. The public engagement group consists of experienced and inexperienced people. We will try to work with the patient advocacy group to ensure that easy-to-understand language abstracts of research results are shared with participants and a wider group of patients. The burden of intervention will be assessed in a comprehensive and reasonable manner through communication between participants and researchers.

The results are not directly communicated to the participants, but the final results will be released. During the follow-up period, each participant will be informed of all examination results.

## Discussion

At present, China has become the country with the largest number of diabetic patients, and T2DM with obesity in China accounts for approximately 60% of all diabetic individuals. The pathogenesis of T2DM is a progressive disorder of islet cells with gradually diminishing effects and decreasing insulin secretion, leading to an increase in plasma glucose [22]. According to relevant studies, obesity can lead to insulin resistance, thus exacerbating the process of T2DM, which is extremely unfavourable to the recovery of patients. For T2DM patients with obesity, obesity should be treated first if T2DM is to be effectively controlled. However, currently, few effective clinical treatment options are available. The usage of sulfonylurea, nateglinide, thiazolidinedione and insulin will increase the weight of patients. Dipeptidyl peptidase-4 inhibitors and α-glucosidase inhibitors are not ideal for weight loss, and sodium-dependent glucose transporter 2 inhibitors are associated with ketoacidosis, urethral mould infection and other risks as well as high medical expenditures [23]. Therefore, in this study, metformin was selected as an intervention drug in patients with good islet function and no contraindication to metformin [24].

However, there are many metabolic disorders in patients with T2DM and obesity. These disorders lead to more complex treatment, requiring increased drug doses or combinations of multiple drugs, resulting in increased adverse reactions and reduced treatment compliance [12]. One of most common disorders is dyslipidaemia. Although statins are the most commonly used lipid-lowering drugs in the clinic, with increased medical research and the expansion of the patient population in recent years, it has been found that long-term use of statins can increase the risk of new diabetes [25] and may affect plasma glucose control in diabetic patients [26].

In clinical practice, doctors need to consider both short-term efficacy and long-term prognosis and comprehensively consider the medical burden. Traditional Chinese medicine (TCM) exhibits the characteristics of safety and reliability in long-term applications and offers multitarget and multipathway control of disease progression. DDG has been prescribed by Longhua Hospital and explored as a famous old Chinese medicine. After approximately 10 years of clinical application, DDG has obtained good clinical curative effects. After treatment, the quality of life of the patient improved. In addition to lowering plasma glucose, the patients lost weight, reduced blood lipids, improved the metabolic disorders of glucose and lipids, and reduced the risk of cardiovascular and cerebrovascular diseases. No serious adverse reactions were observed.

However, there is a lack of large-sample studies for the clinical validation of DDG, and our previous study is a single-centre self-control study, which has some limitations. Therefore, we hope to further objectively evaluate the clinical effectiveness of DDG through a multicentre, double-blind, randomized, controlled study and comprehensively evaluate the benefits of the intervention in patients with T2DM complicated with obesity from the aspects of blood lipids, plasma glucose, insulin resistance and a chronic inflammatory status. In addition, safety indexes, such as routine blood tests, routine urine tests, routine faecal tests, liver and kidney function tests and electrocardiograms, will be monitored before, during and after treatment to evaluate the safety of DDG. Finally, the follow-up period of this study is 24 weeks. Such a long follow-up period is useful to further explore whether weight rebound and index recovery would occur after stopping intervention. This follow-up is useful to objectively and comprehensively evaluate the clinical efficacy and safety of DDGs to provide a new drug choice for clinical treatment that can effectively improve T2DM with obesity.

However, the study design still has some limitations. First, the placebo contains only excipients, such as sucrose, lactose and dextrin, which may have an impact on indicators in T2DM patients with obesity. In addition, DDG is a TCM granule with special odours. Theoretically, the placebo should be consistent with DDG in colour, shape and odour, but it may be difficult to achieve complete consistency in practice. Second, our experiment will be conducted in four centres in the same region of China, and it is uncertain whether similar results will be obtained in other regions and races.

### Trial status

Recruitment will start in 01 June 2021. The current protocol version is 2.0, dated 07 September 2020.

## Data Availability

Not applicable.
